# Thoracoscopy for Esophageal Diverticula After Esophageal Atresia With Tracheo-Esophageal Fistula

**DOI:** 10.3389/fped.2021.663705

**Published:** 2021-05-04

**Authors:** Zhao Yong, Wang Dingding, Hua Kaiyun, Gu Yichao, Zhang Yanan, Liao Junmin, Yang Shen, Li Shuangshuang, Wang Peize, Huang Jinshi

**Affiliations:** Department of Neonatal Surgery, Beijing Children's Hospital, National Center for Children's Health, Capital Medical University, Beijing, China

**Keywords:** esophageal diverticulum, esophageal atresia, tracheoesophageal fistula, thoracoscopic, children

## Abstract

**Background:** Esophageal diverticulum (ED) is an extremely rare complication of congenital esophageal atresia (EA) with or without tracheoesophageal fistula (TEF) surgery. We aimed to investigate feasible methods for the treatment of this rare complication.

**Methods:** We retrospectively reviewed all patients with EA/TEF at Beijing Children's Hospital from January 2015 to September 2019. The clinicopathological features of patients with ED after EA/TEF surgery were recorded. Follow-up was routinely performed after surgery until December 2020.

**Results:** Among 198 patients with EA/TEF, ED only occurred in four patients (2.02%; one male, three female). The four patients had varying complications after the initial operation, including anastomotic leakage (3/4), esophageal stenosis (3/4), and recurrence of TEF (1/4). The main clinical symptoms of ED included recurrent pneumonia (4/4), coughing (4/4), and dysphagia (3/4). All ED cases occurred near the esophageal anastomosis. Patients' age at the time of diverticulum repair was 6.6–16.8 months. All patients underwent thoracoscopic esophageal diverticulectomy (operation time: 1.5–3.5 h). Anastomotic leakage occurred in one patient and spontaneously healed after 2 weeks. The other three patients had no peri-operative complications. All patients were routinely followed up after surgery for 14–36 months. During the follow-up period, all patients could eat orally, had good growth and weight gain, and showed no ED recurrence or anastomotic leakage on esophagogram.

**Conclusions:** ED is a rare complication after EA/TEF surgery and is a clear indication for diverticulectomy. During the midterm follow-up, thoracoscopic esophageal diverticulectomy was safe and effective for ED after EA/TEF surgery.

## Introduction

Congenital esophageal atresia (EA) with or without tracheoesophageal fistula (TEF) is a serious digestive malformation with an incidence of ~1/2000–4500 (1). Esophageal diverticulum (ED) is a diverticulum of one or more layers of the esophageal wall. ED can be divided into two types; the true diverticulum includes the full layers of the esophageal wall, and the pseudo diverticulum only includes the mucosa and submucosa (2). ED is relatively rare in adults, with an incidence of ~0.06–4%, and mainly occurs in the elderly (3). Additionally, ED can occur as an extremely rare complication of EA/TEF surgery. Currently, only a few studies have reported the occurrence of ED after EA/TEF surgery. According to different reports, the incidence of ED is between 0.0% and 1.9% in patients who have undergone EA/TEF surgery (4–6). However, to the best of our knowledge, no reports have described the diagnosis and treatment of ED after EA/TEF surgery.

In this study, we retrospectively analyzed the diagnosis and treatment of patients with ED after EA/TEF surgery at our hospital. The objective of this study was to discuss feasible diagnostic and treatment methods for this rare complication after EA/TEF surgery.

## Patients and Methods

### Data Collection

We retrospectively reviewed all patients with EA/TEF at our institution between January 2015 and September 2019. Patients with ED after EA/TEF surgery were selected, and the clinicopathological features were recorded. Follow-up was routinely performed after surgery. This retrospective study was approved by the ethics committee of Beijing Children's Hospital (approval no. 2019-k-333), and the families of all patients provided written informed consent and agreed to participate in the study.

### Diagnostic Method

Esophageal angiography was used to define the diagnosis of ED after EA/TEF and to evaluate the size and site of ED. Airway endoscopy, digestive endoscopy, and chest computed tomography (CT) were routinely performed to exclude other related diseases before diverticulectomy.

### Surgical Method

**Figure 1:**
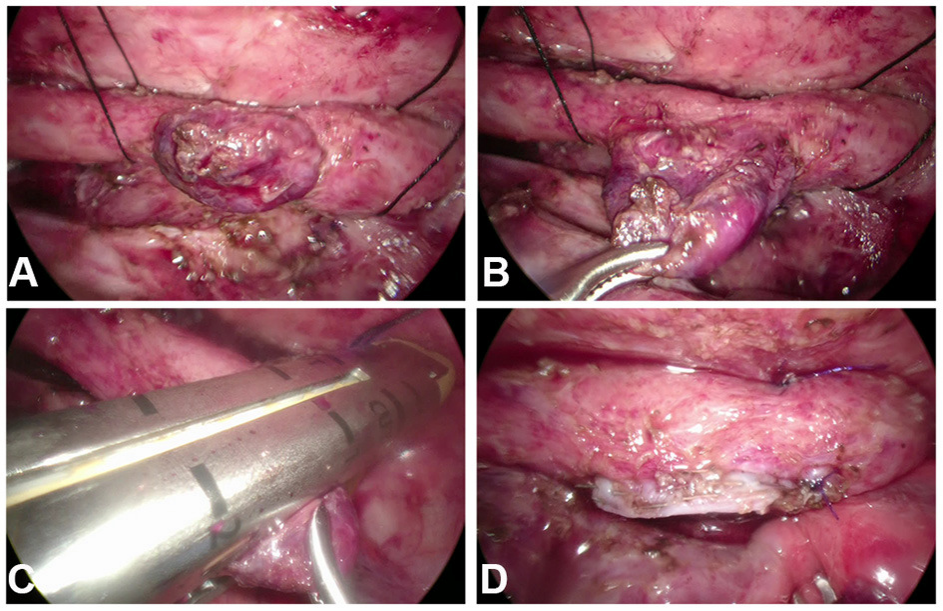
**(A,B)** The neck of the esophageal diverticulum (ED) was fully exposed. **(C)** The linear cutter stapler was positioned at the neck of the ED and close to the esophagus. **(D)** The ED was excised by the stapler.

Thoracoscopic esophageal diverticulectomy was routinely performed from the right side. A 5-mm thoracoscope was placed in the fifth intercostal space of the right infrascapular line. Two 3-mm trocars were placed in the second intercostal space in the mid axillary line and the fifth intercostal space in the posterior axillary line to establish operating channels. The intrathoracic pressure was maintained at 6–8 mmHg. The adhesion between the lung tissue and chest cavity was loosened, and the neck of the ED was fully exposed. The left-hand operating hole replaced with a 10-mm trocar, a surgical linear cutter stapler with a nail length of 30 mm, was inserted, and the diverticulum close to the esophagus was resected (Figure 1). The chest drainage tube was routinely indwelled. After the operation, patients were routinely transferred to the pediatric intensive care unit (PICU) under tracheal intubation.

### Postoperative Follow-Up

Routine esophagogram was performed 7 and 14 days after the operation during hospitalization. Outpatient follow-up and esophagogram were routinely scheduled in the first month, third month and 6 month after surgery. Then outpatient follow-up was scheduled every 3 months during the first 1 year after surgery and every 6 months thereafter. If postoperative esophageal stenosis was found by esophagogram, endoscopy was used to detect and treat the stenosis with endoscopic balloon dilatation. All patients were followed up until the end of December 2020.

## Results

Among the 198 patients with EA/TEF at our institution between January 2015 and September 2019, ED only occurred in four patients (2.02%) during the follow-up period. Of these four patients, one was male, and three were female. EA repair surgery, including open or thoracoscopic ligation of the fistula and end-to-end anastomosis of esophageal segments, was performed in all patients 1 week after birth. The four patients had various complications after the initial operation, including anastomotic leakage (3/4), esophageal stenosis (3/4), and recurrence of TEF (1/4). Two patients also had malformations of other systems, including scoliosis (1/4) and vertebral deformities (2/4). The main clinical symptoms of ED included recurrent pneumonia (4/4), coughing (4/4), and dysphagia (3/4). All diverticula were located near the esophageal anastomosis, between T3 and T6, and all were therefore thoracic ED. Patient age at the time of diagnosing ED ranged from 2.5 to 9.3 months (median age: 6.2 months). The patient age of diverticulum repair was 6.6 to 16.8 months (median age: 9.5 months). By the time of diverticulectomy, the average long diameter of these EDs increased from 15.2 to 22.2 mm. Body weight at the time of operation was 6.5–9.5 kg (median weight: 7.3 kg). All four patients underwent a thoracoscopic esophageal diverticulectomy. Owing to recurrent TEF, patient 4 underwent thoracoscopic TEF resection at the same time. The operation time ranged from 1.5 to 3.5 h (average: 2.2 h). In all patients, intra-operative bleeding was <5 mL, and no blood transfusions were required during the operation. After the operation, patients were routinely transferred to the PICU. The postoperative time of invasive ventilator use was 1–7 days (average: 3 days). The postoperative hospital stay time was 10–20 days (average: 14 days). In patient 4, anastomotic leakage occurred 1 week after surgery, as observed by esophagogram. After enteral nutrition via a nasojejunal tube and conservative supportive treatment were performed, the anastomotic leakage healed after 2 weeks, as demonstrated by radiography. The other three patients had no peri-operative complications. Clinicopathological data for the four patients with ED after EA/TEF are shown in Table 1.

**Table 1:** Clinicopathological features of patients with esophageal diverticulum after esophageal atresia/tracheoesophageal fistula surgery.

**ID**	**Gender**	**Age of diagnosis (months)**	**Age of surgery (months)**	**Body Weight (kg)**	**Symptoms**	**Long diameter (mm)**	**Site**	**Complications after the initial surgery**	**Combined deformity**	**Operation time (h)**	**Tracheal intubation time (d)**	**Postoperative hospital stay**	**Postoperative complications**	**Postoperative pathology**
1	Female	2.5	6.6	6.5	Coughing, recurrent pneumonia	25.5	T3-5	Anastomotic leakage	None	2	3	12	None	Pseudodiverticulum
2	Female	6.2	9.5	8.0	Dysphagia, Coughing, recurrent pneumonia	22.1	T4-5	Esophageal stricture	Scoliosis, vertebral deformities	1.5	1	10	None	True diverticulum
3	Female	6.8	13.1	9.5	Dysphagia, Coughing, recurrent pneumonia	24.6	T4-6	Anastomotic leakage, esophageal stricture	None	1.5	5	14	None	Pseudodiverticulum
4	Male	9.3	16.8	6.0	Dysphagia, Coughing, recurrent pneumonia	16.7	T4-5	Anastomotic leakage, esophageal stricture, recurrent TEF	Vertebral deformities	3.5	7	20	Anastomotic leakage, esophageal stricture	Pseudodiverticulum

*ED, esophageal diverticulum; EA, esophageal atresia; TEF, tracheoesophageal fistula*.

All patients were routinely followed up after surgery for 14–36 months. During the follow-up period, all patients were able to eat by mouth and had good growth and satisfactory weight gain, without coughing, pneumonia, or dysphagia. Additionally, esophagogram revealed no ED recurrence or anastomotic leakage.

### Illustrative Cases

Patient 1 was a 6.6-month-old girl who was diagnosed with congenital EA type IIIa after birth. Five days after birth, the patient underwent thoracoscopic ligation of the fistula and end-to-end anastomosis of the esophageal segments. Esophagogram indicated leakage of esophageal anastomosis at 2 weeks after surgery, which healed after conservative treatment. At 2.5 months, the patient visited our outpatient clinic for choking and repeated pneumonia. Esophagogram revealed a 14.8 mm diverticulum at the right posterior edge of the esophagus. The symptoms were not significantly relieved by conservative treatment. The esophagus re-examination 6 months after birth showed that the long ED diameter increased to 25.5 mm, compared to its initial examination. Airway endoscopy and digestive endoscopy showed that the ED did not communicate with the trachea; chest CT was performed to exclude inflammatory consolidation of the lung. The patient underwent thoracoscopic esophageal diverticulectomy, and postoperative pathology revealed a pseudodiverticulum, which only contained mucosa and submucosa tissues without muscle tissue involvement. After the operation, the coughing and pneumonia symptoms disappeared, and the patient was gradually able to consume a normal diet. The patient was followed up for 23 months without symptoms.

Patient 2 was a 9.5-month-old girl diagnosed with congenital EA type IIIb and underwent thoracotomy ligation of the fistula and end-to-end anastomosis esophageal segments 5 days after birth. At 2 months, the patient had recurrent TEF. After conservative treatment and enteral nutrition for 2 months, she underwent thoracoscopic ligation of recurrent TEF 4 months after birth. In 6.2 months, the patient had dysphagia, and the esophagogram revealed esophageal stenosis and an ED at the upper edge of the esophageal stenosis. Then, she underwent endoscopic balloon dilatation three times, which was expanded to 10 mm. However, the patient still exhibited choking and repeated pneumonia. Esophagogram and endoscopy revealed that the ED increased from 15.7 mm to 22.1 mm. The patient underwent thoracoscopic esophageal diverticulectomy, and postoperative pathology revealed a true diverticulum, which contained a thickened muscle layer in the diverticula wall. After the operation, the symptoms of coughing and pneumonia disappeared. The patient was followed up for 33 months without symptoms.

**Figure 2:**
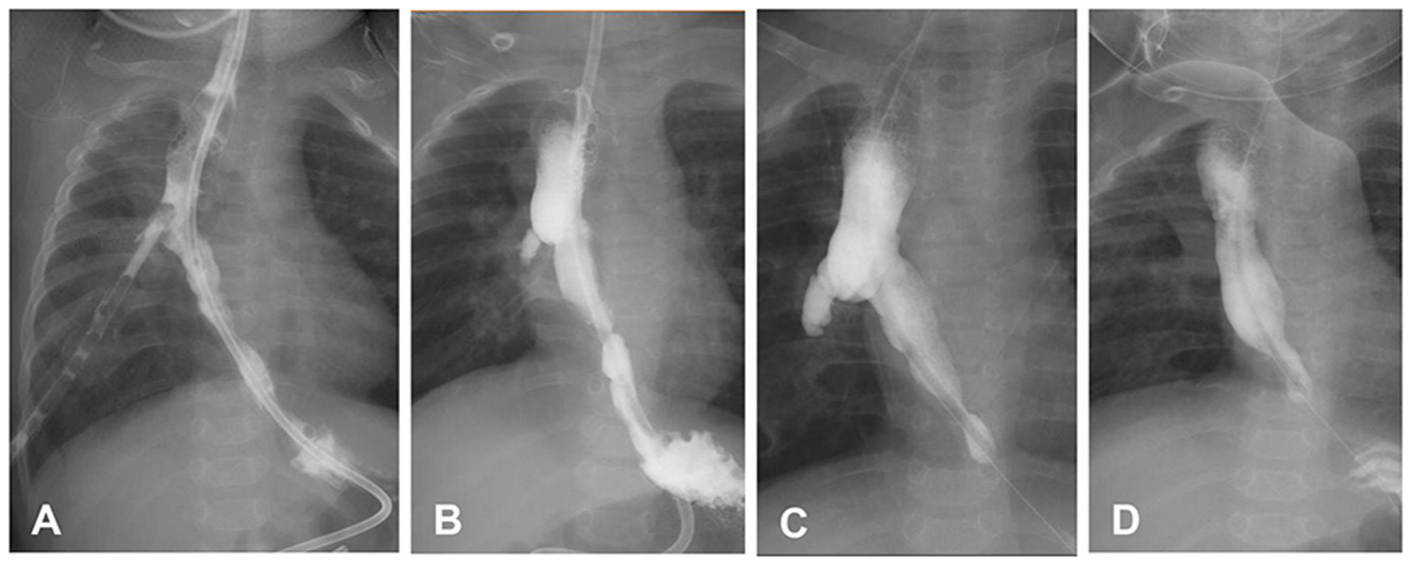
Changes in the esophageal diverticulum (ED) of patient 3. **(A)** After esophageal atresia/tracheoesophageal fistula surgery, the contrast agent flowed into the chest drainage tube through the esophageal anastomosis leakage. **(B)** The thoracoscopic drainage tube was removed prematurely, and the ED appeared. **(C)** The ED was enlarged. **(D)** Two weeks after diverticulectomy, the esophagus was in good shape. In C and D, the patient underwent laparoscopic fundoplication, leading to postoperative distal esophageal stenosis.

Patient 3 was a 13.1-month-old girl. Three days after birth, the patient underwent thoracotomy ligation of the fistula and end-to-end anastomosis of the esophageal segments due to congenital EA type IIIb. Anastomotic leakage occurred 2 weeks after surgery and healed after conservative treatment of 4 weeks. At 6.8 months of age, the patient exhibited repeated vomiting, choking, and pneumonia. Esophagogram showed gastroesophageal reflux and right-side ED at the T4–6 level (Figure 2). The 24-h pH test confirmed pathological reflux in the lower esophagus. At 8 months of age, she underwent laparoscopic fundoplication to improve gastroesophageal reflux. After the operation, coughing and repeated pneumonia symptoms were still observed, and endoscopic balloon dilatation of the esophageal stenosis was performed several times, gradually expanding to 12 mm. She still had repeated pneumonia and choking, and re-examination of esophagogram and endoscopy revealed that the ED increased from 17.1 mm to 24.6 mm. Thoracoscopic esophageal diverticulectomy was performed, and the pathological response showed no muscle layer in the diverticula wall, indicating the presence of pseudodiverticulum. After the operation, the patient's coughing symptoms improved, and she gradually returned to a normal diet. The patient was followed up for 36 months without symptoms.

**Figure 3:**
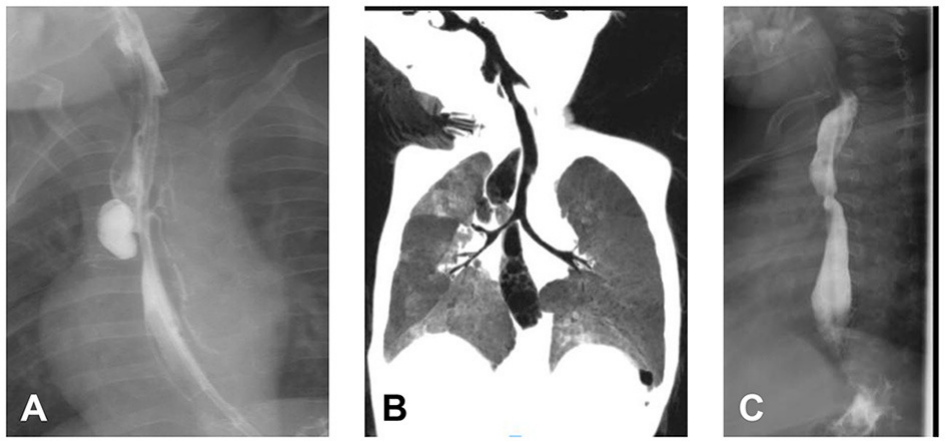
The pre-operative and postoperative radiography of patient 4. **(A)** Esophagogram revealed esophageal diverticulum (ED) (on the right side of the esophagus) combined with recurrent tracheoesophageal fistula (on the left side of the esophagus). **(B)** ED on chest computed tomography. **(C)** Two weeks after diverticulectomy.

Patient 4 was a 16.8-month-old boy with congenital EA type IIIb who underwent thoracotomy ligation of the fistula and end-to-end anastomosis of esophageal segments 3 days after birth. Postoperative esophageal anastomotic leakage occurred, and complication was alleviated after conservative treatment. Recurrent TEF was diagnosed 5 months after birth, and conservative treatment for pneumonia and enteral nutrition were performed. In 9.3 months, esophagogram and endoscopy revealed an ED on the right side of the esophagus, except for recurrent TEF and esophageal stenosis (Figure 3). Then, he underwent thoracoscopic ligation of recurrent TEF. However, he still had repeated pneumonia and choking after surgery. At 16 months after birth, esophagogram showed the ED increased from 13.0 mm to 16.7 mm and a novel recurrent TEF on the left side. Thoracoscopic ligation of the recurrent TEF and esophageal diverticulectomy were performed. Pathological examination showed no muscle layer in the diverticula wall, indicating pseudodiverticulum. One week after surgery, anastomotic leakage occurred by esophagogram, which healed spontaneously after conservative treatment for 1 week. Two months postoperatively, the patient underwent endoscopic balloon dilatation due to esophageal stenosis, which gradually expanded to 12 mm. He slowly returned to his normal diet after the operation and was followed up for 14 months without symptoms.

## Discussion

ED is an extremely rare complication of EA/TEF surgery. Myers et al. reported that among 498 patients with EA/TEF after surgery, the incidence of ED was only 0.2% (6). Taghavi et al. found that among 56 patients with H-type treated by surgery, the incidence of ED was 1.9% (5). Additionally, in a study evaluating the postoperative complications of 92 patients with EA/TEF, ED did not occur in any of the 92 patients after surgery (4). Among the 198 patients with EA/TEF at our institution, ED only occurred in four patients after surgery, with an incidence rate of 2.02%. This was consistent with the results of these previous studies, confirming that ED was a rare complication after EA/TEF surgery.

According to its pathogenesis, ED is divided into two types, i.e., pulsion diverticulum and traction diverticulum. Most ED cases are pulsion diverticulum, which is related to esophageal dysmotility, functional or mechanical obstruction, or weakness of the esophageal wall. The esophageal sphincter cannot relax normally, resulting in compression of the esophageal cavity and forcing the mucosa and submucosa to herniate through the esophageal muscles (pseudodiverticulum). Traction diverticulum is relatively rare. The inflammatory reaction around the esophagus causes scarring of the esophageal wall, and local traction of the esophageal wall leads to the formation of a diverticulum (true diverticulum). The primary pulsion diverticulum commonly occurs at the proximal and distal ends of the esophagus and includes Zenker diverticulum and epiphrenic diverticulum. In contrast, traction diverticulum, including thoracic ED, commonly occurs in the middle of the esophagus (2, 7, 8). In all four patients in our series, ED was considered thoracic ED; however, the pathogenesis of ED after EA/TEF surgery is different from that of the primary ED. Our four patients had various complications after the initial operation. For example, anastomotic leakage occurred in three patients after the initial surgery, and the pathological results of these three cases after diverticulectomy showed no muscle structures involved, suggestive of pseudodiverticulum. The formation mechanism in these cases may be related to continuous saliva and esophageal secretions through the anastomosis, resulting in persistent pleurisy and local inflammation around the esophagus and thereby causing the formation of cavities and local fibrosis. Moreover, the distal esophagus combined with stenosis resulted in increased pressure in the proximal esophagus, forcing the mucosa and submucosa to herniate via the anastomotic fistula to form the ED. The pathology results for patient 2 after diverticulectomy revealed the involvement of muscular structures, indicating true diverticulum. The patient did not experience anastomotic leakage after surgery; however, recurrent TEF occurred in the patient after 2 months. In the second surgery for recurrent TEF, the fistula was ligated close to the trachea, resulting in an excessively long esophageal fistula. Additionally, these features combined with anastomotic stricture of the esophagus led to increased pressure in the proximal esophageal cavity, and the stump of the esophageal fistula gradually expanded to form a diverticulum. Livaditis esophageal myotomy can lead to ED after EA/TEF surgery (9). None of the children in our study underwent Livaditis esophageal myotomy during the initial operation. The pathogenesis of ED after EA/TEF surgery was different from that of the primary ED and may be related to esophageal anastomosis leakage, anastomotic stricture or the long esophageal fistula stump, especially for patients with multiple complications. For patients who had anastomosis leakage after surgery, early and aggressive endoscopic balloon dilatation for preventing anastomotic stricture might help in reducing the occurrence of ED. However, further studies are still needed to elucidate the detailed pathogenesis of this condition.

The common symptoms of thoracic ED in adults include dysphagia, gastroesophageal reflux, heartburn, aspiration pneumonia, and weight loss (10, 11). In our four patients, the main symptoms included recurrent pneumonia, dysphagia, coughing, and unsatisfactory weight gain. These symptoms were similar to the common symptoms of some complications after EA/TEF surgery, such as esophageal stenosis, gastroesophageal reflux, and recurrent TEF. Esophagogram is the preferred diagnostic method for ED; this approach can be used to confirm the diagnosis and evaluate the location and size of ED (12). Airway endoscopy, digestive endoscopy, and chest CT can be used to exclude other related diseases, such as recurrent TEF and inflammatory consolidation of the lung. Measurement of esophageal pressure is the gold standard for diagnosing esophageal dyskinesia. Studies have shown that more than 90% of ED patients may have abnormal esophageal motility; however, it is still unclear whether esophageal dyskinesia was the cause of ED or a symptom of ED (13). Some scholars have suggested that esophageal dyskinesia is always present in patients with ED, indicating that pressure measurement is not required (14). Gastroesophageal reflux is closely related to ED, and 24-h esophageal pH monitoring can confirm gastroesophageal reflux and guide surgical or drug treatment (12). All four patients underwent esophageal angiography to confirm the diagnosis of ED. All patients underwent airway endoscopy, digestive endoscopy, and chest CT, and recurrent TEF was diagnosed in patient 4. Moreover, 24-h esophageal pH monitoring was used to confirm the symptoms of gastroesophageal reflux in patient 3, which indicated pathological reflux, and laparoscopic fundoplication was performed. Esophagogram was a preferred diagnosis method for patients with ED after EA/TEF surgery. Moreover, airway endoscopy, digestive endoscopy, and chest CT should be performed to exclude surgical contraindications. Esophageal pH monitoring is a useful examination method for symptomatic patients. Comprehensive examination could contribute to the evaluation and provide direction for subsequent surgical approaches.

In adult thoracic ED, most surgeons agreed to surgical intervention in symptomatic patients; however, the treatment of patients with the mild or asymptomatic disease remains controversial (15–19). Altorki et al. reported that aspiration occurred in nine of 20 adult patients, and three experienced life-threatening complications (20). They strongly supported surgical intervention for all patients. Macke et al. summarized 15 years of experience in the treatment of thoracic ED in adults and concluded that surgical intervention should be carried out for patients with asymptomatic large diverticula or abnormal esophageal motility; additionally, asymptomatic patients, particularly those with a high risk of surgery, should be closely monitored (11). All four patients in this series had obvious clinical symptoms. Because infants and young children are more prone to aspiration, coughing, and other symptoms, we recommend that active surgical intervention be performed for mild or even asymptomatic ED after EA/TEF. Notably, anastomotic stricture should be treated aggressively before esophageal diverticulectomy, which might be the cause of ED.

The surgical treatment of thoracic ED mainly included open surgical approaches and minimally invasive approaches. With advancements in minimally invasive techniques, the surgical linear cutter stapler has become widely used in pediatric gastrointestinal surgery (20–22). All four patients in this group underwent thoracoscopic esophageal diverticulectomy, and we identified four technical details related to the surgery. First, it was necessary to fully expose the neck of the diverticulum during the separation of the diverticula. The surgical linear cutter stapler must be inserted close to the esophagus to avoid leaving a residual diverticulum. Second, placing a gastric tube could help stabilize the esophagus and prevent esophageal cavity stricture. Third, the muscles or pleura around the staple line of the esophagus should be freed to reduce the incidence of anastomotic leakage. Finally, three of the four patients in this series had pseudodiverticulum, and the diverticula wall had no muscle tissue. The cutter stapler might be useful to cut the pseudodiverticulum but not hand-tied ligature. Additionally, using a cutter stapler could reduce the operation time and the incidence of postoperative anastomotic leakage. None of the patients in the study underwent transit thoracotomy. Anastomotic leakage and esophageal stricture occurred in one patient 1 week after surgery; this symptom was alleviated by conservative treatment and balloon dilatation. The other three patients had no peri-operative complications. During the follow-up period, none of the patients had any other complications. Thoracoscopic esophageal diverticulectomy proved to be a reliable and effective treatment method for ED after EA/TEF, but must be performed by surgeons experienced in open and minimally invasive esophageal surgery.

Currently, the use of routine or selective esophageal Heller's myotomy during diverticulectomy for ED in adults is controversial. Studies have reported that the incidence of esophageal dyskinesia in adults with ED is 45–100% (23–27). Simple mechanical obstruction or combined functional obstruction could lead to increased pressure in the esophageal cavity and the formation of the ED. Therefore, some surgeons tend to routinely perform Heller's myotomy, regardless of the presence or absence of distal esophageal dyskinesia (25). In contrast, other researchers have been more inclined to perform Heller surgery when distal esophageal dyskinesia is clearly diagnosed before surgery (18). Because the pathogenesis of ED after EA/TEF is different from that of adult ED, the main factor resulting in increased pressure in the esophageal cavity was esophageal stenosis, which could be eliminated by endoscopic balloon dilation. Therefore, we recommend that ED after EA/TEF surgery may not require routine Heller surgery. None of the four patients in this study underwent Heller surgery. During the follow-up period, none of the patients had shown symptoms such as distal esophageal stenosis or recurrence of ED. However, long-term follow-up is still needed to assess this issue.

However, the study also has several limitations. Due to the rarity of this complication, the number of patients is small, which is not suitable for statistical analysis. The possible risk factors for ED after EA/TEF still need to be confirmed by larger samples. Additionally, the study is a single-center retrospective research, and more clinical prospective studies are required to confirm our results.

In summary, ED is a rare complication after EA/TEF surgery and is a clear indication for diverticulectomy. Our results showed that thoracoscopic esophageal diverticulectomy was safe and effective for ED after EA/TEF surgery during mid-term follow-up. Further follow-up is needed to assess the reliability of these results. Additionally, surgical treatment is suggested to be performed by surgeons with extensive thoracoscopic experience.

## Data Availability Statement

The original contributions presented in the study are included in the article/supplementary material, further inquiries can be directed to the corresponding author.

## Ethics Statement

The studies involving human participants were reviewed and approved by the ethics committee of Beijing Children's Hospital. Written informed consent to participate in this study was provided by the participants' legal guardian/next of kin. Written informed consent was obtained from the individuals, and minors' legal guardian/next of kin, for the publication of any potentially identifiable images or data included in this article.

## Author Contributions

ZYo, WD, and HJ contributed to conception and design of the study. GY analyzed the data. HK and LJ processed the figures. ZYo and WD wrote the first draft of the manuscript. LS, WP, and ZYa wrote sections of the manuscript. All authors contributed to the article and approved the submitted version.

## Conflict of Interest

The authors declare that the research was conducted in the absence of any commercial or financial relationships that could be construed as a potential conflict of interest.
